# Composition and function of the Galapagos penguin gut microbiome vary with age, location, and a putative bacterial pathogen

**DOI:** 10.1038/s41598-023-31826-y

**Published:** 2023-04-01

**Authors:** Sage D. Rohrer, Gustavo Jiménez-Uzcátegui, Patricia G. Parker, Lon M. Chubiz

**Affiliations:** 1 Department of Biology and Whitney R. Harris World Ecology Center, University of Missouri-St. Louis, One University Blvd., St. Louis, MO 63121 USA; 2 Charles Darwin Research Station, Charles Darwin Foundation, Galapagos, Ecuador; 3 WildCare Institute, Saint Louis Zoo, One Government Drive, St. Louis, MO 63110 USA

**Keywords:** Microbial ecology, Ecology

## Abstract

Microbial colonization plays a direct role in host health. Understanding the ecology of the resident microbial community for a given host species is thus an important step for detecting population vulnerabilities like disease. However, the idea of integrating microbiome research into conservation is still relatively new, and wild birds have received less attention in this field than mammals or domesticated animals. Here we examine the composition and function of the gut microbiome of the endangered Galapagos penguin (*Spheniscus mendiculus*) with the goals of characterizing the normal microbial community and resistome, identifying likely pathogens, and testing hypotheses of structuring forces for this community based on demographics, location, and infection status. We collected fecal samples from wild penguins in 2018 and performed 16S rRNA gene sequencing and whole genome sequencing (WGS) on extracted DNA. 16S sequencing revealed that the bacterial phyla Fusobacteria, Epsilonbacteraeota, Firmicutes, and Proteobacteria dominate the community. Functional pathways were computed from WGS data, showing genetic functional potential primarily focused on metabolism—amino acid metabolism, carbohydrate metabolism, and energy metabolism are the most well-represented functional groups. WGS samples were each screened for antimicrobial resistance, characterizing a resistome made up of nine antibiotic resistance genes. Samples were screened for potential enteric pathogens using virulence factors as indicators; *Clostridium perfringens* was revealed as a likely pathogen. Overall, three factors appear to be shaping the alpha and beta diversity of the microbial community: penguin developmental stage, sampling location, and *C. perfringens*. We found that juvenile penguins have significantly lower alpha diversity than adults based on three metrics, as well as significantly different beta diversity. Location effects are minimal, but one site has significantly lower Shannon diversity than the other primary sites. Finally, when samples were grouped by *C. perfringens* virulence factors, we found dramatic changes in beta diversity based on operational taxonomic units, protein families, and functional pathways. This study provides a baseline microbiome for an endangered species, implicates both penguin age and the presence of a potential bacterial pathogen as primary factors associated with microbial community variance, and reveals widespread antibiotic resistance genes across the population.

## Introduction

Resident microbes diversely affect animal health. Some long-term members of the microbiome may facilitate digestion or provide immune system training for the host^1^. Colonization resistance is an important benefit, as mutualistic microbes may out-compete a pathogenic invader for space and nutrients or even release toxins as a deterrent^2^. Some community members may also be opportunistically pathogenic, occupying an inconspicuous position in the microbiome until a disruption occurs, then infecting the host^3,4^.

Characterizing the normal microbiome for species of concern provides an essential baseline from which to measure changes. This has the potential to help zoos improve their level of care for collection animals as well as to increase reintroduction success by mimicking a species’ normal microbiome^5,6^. However, microbial community assessments can also improve management of wild populations in a number of ways. Dysbiosis in the microbiome (i.e. disruption of the normal bacterial community) can be an indicator of health problems and is associated with a number of diseases^1,7^. Metagenomic assessments can reveal potential pathogens that may pose a threat to the population^8^. Sequencing data can additionally be used to assess the level of antimicrobial resistance in the community, known as the “resistome,” which at high levels can indicate a potentially dangerous level of connectivity between humans and wildlife^9^. Thus, microbiome research may provide a critical perspective for conservation efforts.

Factors structuring these resident microbial communities are complex and can vary substantially between taxa. Diet is known to be a primary driver of microbiome composition and taxa with atypical diets tend to have distinct microbiomes^10–12^. For instance, the vampire finch (*Geospiza septentrionalis*) supplements its diet in an unusual way by eating eggs, guano, and the blood of larger birds, and the species also exhibits a unique microbiome profile compared to the other Darwin’s finches^13^. These dietary changes can be rapid—one study of human diet found that switching from a plant- to animal-based diet resulted in reduced carbohydrate fermentation and increased protein fermentation by gut microbes in a matter of days, with corresponding abundance changes in bacteria associated with those activities^14^. Environmental factors such as season and habitat are known to affect gut microbiomes as well, though these differences are largely attributed to changes in food availability between seasons^15,16^. However, diet is a better predictor for microbiome composition and function in mammals than it is for birds, and the effect of diet is weakest in the microbiomes of both bats and flying birds, possibly due to the shorter intestines associated with flight adaptation^17^.

Demographic factors such as the host’s sex or developmental stage can also play a role in shaping the microbiome^18,19^. One study found that cloacal microbiomes differed between male and female rufous-collared sparrows (*Zonotrichia capensis*) during breeding seasons, with the male microbiome becoming more diverse at the onset of the breeding season^19^. Hormonal differences or immune variation between sexes may be responsible to some degree^19–21^. Many taxa also undergo microbiome changes throughout development; for example, little penguins (*Eudyptula minor*) exhibit increased abundances of Firmicutes and Bacteroidetes as they mature^22^. Microbial community differences based on sex and age are not consistent across taxa, and in some cases the effect size is quite small compared to other factors, indicating a continued need to assess community drivers on a case-by-case basis in wild populations^23,24^.

Once the normal microbiome is understood for a given species, monitoring the microbiome using fecal samples may be a valuable non-invasive assay for wild populations of concern^25,26^. Scat microbiome assays are being developed to provide insights into population demographics and host health. For example, one study in Rocky Mountain elk (*Cervus canadensis*) determined microbial predictors for host age, sex, body fat, and biogeography, reliably classifying individuals based on fecal microbiome samples^25^. Microbiome diversity (i.e. the number of species in a microbial community) can also indicate vulnerability to disease—for example, juvenile ostriches with initially low bacterial diversity can be more likely to develop pathogen-associated dysbiosis and later succumb to enterocolitis mortality^7^. Testing the fecal microbiome can also reveal anthropogenic influence on a wild population, often through dysbiosis in the microbial composition and abundance^26,27^. Human influence may also be detected through the increased presence of antibiotic resistance genes in the microbiome; human-associated factors such as wastewater or livestock can introduce resistance genes to new environments, where the resistance genes can be rapidly disseminated through microbial communities via horizontal gene transfer^9,28^. However, many wild microbiome studies rely solely on 16S rRNA gene sequencing instead of shotgun sequencing, which provides little indication of pathogenicity or antibiotic resistance genes in a microbiome^29^. Widespread use of high-resolution metagenomic data across wild microbiome studies and the validation of microbial assays are necessary steps before microbiome-based tools can be used to make management recommendations for wild populations^5,25^.

This study examines the gut microbiome of the Galapagos penguin (*Spheniscus mendiculus*), a highly range-restricted species which occurs only in the Galapagos Islands^30^. The Galapagos penguin forages near the shore, consuming schooling fish (such as mullets and sardines) and crustaceans^31–33^. This penguin faces regular population bottleneck events when the nutrient-rich Cromwell Current is disrupted periodically by warm El Niño weather patterns, leading to reduced fish availability and ultimately penguin starvation^33^. The species is classified as endangered by the International Union for Conservation of Nature (IUCN) due to the severe declines associated with these events as well as their highly restricted range^30,33^. The frequent population bottlenecks are likely the reason for the genetic homogeneity found in this species—both microsatellite markers and major histocompatibility complex (MHC) sequences demonstrate a low degree of genetic variation^34–36^.

The low genetic variation may leave the penguin population vulnerable to introduced diseases^37,38^. Previous studies have found evidence of prior infections by *Chlamydophila psittaci* and *Toxoplasma gondii*^39,40^, as well as infections by microfilariae (species unknown) and a lineage of *Plasmodium* (Lineage A)^38,41^*.* However, microbiome characterization and a thorough assessment of enteric pathogens using high-throughput sequencing tools has not been completed and would provide valuable insight into the health of this species. Furthermore, widespread antibiotic resistance genes have been found previously in the Galapagos Islands^9,42,43^, but the extent to which antibiotic resistance is associated with the Galapagos penguin was unknown as we began this study.

This research thus has four goals: (1) establish a baseline for gut microbiome taxonomy and function in the Galapagos penguin, (2) identify putative enteric pathogens, (3) characterize the resistome; and (4) explore drivers of community structure. We hypothesized that hormonal variation and contrasting foraging habits may lead to distinctive microbiomes, and so microbial community structure would vary depending on both sex and age, respectively. Due to the largely homogeneous environment of the western coast of Isabela Island, we hypothesized that different locations may be a minimal factor in gut microbiome communities, particularly since the penguin population shows significant movement between sites and the penguins tend to forage near the coast^32,36^. Finally, we hypothesized that gut pathogens -if found- may be associated with community changes facilitated through either disruptive or opportunistic invasion.

## Methods

All Galapagos penguin fecal samples used in this study were collected in July of 2018 from Isabela Island and the Marielas Islets in Galapagos, Ecuador. Sample collection methods were performed in accordance with relevant guidelines and regulations; this study was approved by the University of Missouri-St. Louis Institutional Animal Care and Use Committee, United States Department of Agriculture, Galapagos National Park Directorate, the Agency for the Regulation and Control of Biosecurity and Quarantine for Galapagos, and the Ecuadorian Ministry of the Environment and Water. Penguins were sampled at three sites on Isabela and the Marielas, with two sample days per site. To collect samples, wild penguins were safely captured on land at each site using long-handled nets and brought to a large boat for processing. Processing included morphological measurements and opportunistic fecal collection. The fecal collection procedure involved harvesting feces from clean plastic sheets placed beneath the penguins during transport and handling, immediately following capture. Fecal samples were preserved in 95% ethanol at room temperature^44^. Males and females were identified based on bill depth measurements and size^45,46^. Each penguin was tagged with a Passive Integrated Transponder tag in the web of one foot (as part of a separate study), enabling the identification of recaptured individuals. For this study, each sample corresponds to an individual penguin. Fecal DNA was extracted within four weeks of sample collection using Qiagen PowerFecal DNA extraction kits (Qiagen, LLC, Germantown, Maryland). Manufacturer instructions were followed for the extraction protocol. DNA was extracted from 0.1 g of feces from each sample (total feces for each sample ranged from 0.25 to 1000 g) based on manufacturer suggestions for avian fecal samples, and homogenization was performed using a vortex adapter at maximum speed for 10 min. DNA was quantified using a Qubit fluorometer. Many samples had low DNA yield (less than 20 ng of total DNA), despite multiple extraction attempts, and only 40 samples with total yield higher than 20 ng were used for sequencing. Extracted DNA was stored at − 20 °C.

### Targeted sequencing

Sequencing of the 16S rRNA gene V4 region was performed with Illumina MiSeq on 40 of the DNA samples, excluding samples with insufficient DNA (< 20 ng), at the University of Michigan Medical School Microbiome Core. The Microbiome Core used the dual indexing sequencing strategy and primers described in Kozich et al. 2013^47^. The V4 region was selected because its length of 250 bp allows forward and reverse reads to fully overlap when sequenced with the cost-efficient Illumina platform, reducing the error rate^47^. Method standardization also facilitates comparisons between microbiome studies, and the V4 region has widespread use, notably through the Earth Microbiome Project and the Mothur Standard Operating Procedure (SOP)^47,48^. Negative sampling and extraction controls were included to assess contamination during processing, and a water sample and a mock community (ZymoBIOMICS Microbial Community Standard) were added by the Microbiome Core prior to library preparation as negative and positive PCR amplification and sequencing controls, respectively. Seven penguin samples were later resequenced alongside a ZymoBIOMICS Gut Microbiome Standard, which had been stored in 95% ethanol prior to extraction to serve as a positive control for the preservation/extraction methods. The resequenced samples were examined to ensure bacterial composition was comparable to the originally sequenced samples; however, all data included in the downstream analysis was generated from the first sequencing run to avoid any potential batch effects. The ZymoBIOMICS Microbial Community Standard was also added to this second sequencing run by the Microbiome Core as a sequencing positive control.

Read analysis was conducted in Mothur, following the Schloss MiSeq standard operating procedure^47,49^. Reads were filtered by base quality and length, aligned with the SILVA 132 reference database^50^ and filtered for alignment quality and chimeras. Samples with less than 10,000 reads following data cleaning steps were excluded, leaving 34 samples. Operational taxonomic unit (OTU) clustering was based on a 97% similarity threshold. Unclassified reads and reads that matched mitochondria, chloroplasts, or archaea were filtered out. Filtered reads were subsampled in Mothur to match the sample with the lowest read count (11,295 reads). Community data were imported into R version 3.6.3 to analyze alpha and beta diversity^51^.

### Whole genome sequencing

Whole genome sequencing (WGS) was performed on 20 of the extracted fecal samples by the University of Michigan Microbiome Core using the Illumina Nextera DNA Flex kit^52^. Four of these samples were later resequenced with the ZymoBIOMICS Gut Microbiome Standard (Zymo Research, Irvine, CA) included in the same run as a positive control to ensure comparable results—the resequenced samples were examined to make sure the bacterial compositions were similar to the original sequence results, but downstream analysis included only data generated in the initial sequencing run. Quality control was performed by trimming reads using Trimmomatic with a sliding window of 4, minimum quality score of 20, and minimum length of 70^53^. These cleaned reads were used directly for read mapping with KMA (k-mer alignment) against two databases, VFDB (Virulence Factor Database) and CARD (Comprehensive Antibiotic Resistance Database), on the platform PATRIC (Pathosystems Resource Integration Center)^54–56^. They were categorized taxonomically using the k-mer based program Kraken 2, also available on PATRIC^57^.

The trimmed reads were also assembled with SPAdes, using the recommended K-mer lengths of 21, 33, 55, 77, 99, and 127, with the BayesHammer module for error correction enabled^58,59^. SPAdes output was assessed with MetaQuast^60^. Compared to single genome assemblies, lower quality is expected for metagenomic assemblies, but two samples with the worst assemblies were ultimately excluded from much of the functional analysis due to poor downstream annotation results; alternate tools relying on reads rather than contigs were similarly problematic for those two samples. The contigs from the SPAdes assembly were classified taxonomically using Kraken 2 on PATRIC.

Finally, parallel read-based and assembly-based approaches were used to achieve a robust picture of the functional profile in these communities. Cleaned reads were categorized functionally using the program HUMAnN 3.0, while contigs were annotated using the program MetaErg^52,61^. Pfam (Protein Family Database) annotations were obtained through both pipelines, which notably annotated virulence-associated proteins in addition to the virulence factors obtained via PATRIC^62^. Pathways were only computed for a minority of gene families in both HUMAnN 3.0 and MetaErg, leaving a majority unclassified. Protein families and metabolism-related KEGG (Kyoto Encyclopedia of Genes and Genomes) pathways detected from the metagenomic assemblies were imported to R to examine differences in metabolic profiles between groups (sex, age, and sampling location)^51,63–65^. Protein family abundances and pathway abundances were subsampled to match the lowest sample (22,259 and 19,684 reads, respectively), after excluding one sample with insufficient protein families and two samples with insufficient pathway results, to allow comparisons between samples without depth-biased results.

### Control assessment

Since negative controls contained ≤ 30 reads following batch filtering steps for the 16S dataset, and < 15 classified fragments following Kraken 2 classification of trimmed shotgun sequencing reads, potential contamination (from sampling, extraction kits, sequencing crossover, etc.) was considered negligible. 16S sequences from the Zymo preservation/extraction control (ZymoBIOMICS Gut Microbiome Standard) were mapped to manufacturer-provided reference sequences using the “seq.error” function in Mothur, which calculated an error rate of 0.0079%. Sequences from the Zymo sequencing control (ZymoBIOMICS Microbial Community Standard) were mapped to 16S reference sequences provided by the University of Michigan Microbiome Core, with an error rate of 0.015% and 0.005% for the first and second sequencing runs. All expected bacterial species with theoretical abundances > 0.01% were detected in the mock communities following processing; some abundance skew was apparent, but this is unlikely to affect the reported results as all study samples were processed in the same batch and comparable to each other. Shotgun sequences of the ZymoBIOMICS Gut Microbiome Standard were directly mapped to the whole genome reference sequences provided by the manufacturer using Bowtie2^66^. Reads successfully mapped to all expected bacteria, archaea, and yeasts.

### Diversity calculations and statistical analysis

Alpha diversity was calculated for each sample from the subsampled 16S rRNA gene sequencing data using the R package phyloseq for three metrics: observed richness, Simpson’s Index, and Shannon Diversity Index^67^. Significant differences based on alpha diversity values were assessed between groups using Kruskal Wallis tests^51^. Two common measures of beta diversity are Jaccard distance, which relies on presence/absence data, and Bray–Curtis dissimilarity, which includes abundance data^68^. Both Jaccard distance and Bray–Curtis dissimilarity were initially used to calculate beta diversity, with similar results; Jaccard distance was ultimately used for this analysis. Beta diversity (Jaccard distance) was calculated in the phyloseq package for both 16S and WGS datasets, and differences in beta diversity were assessed using permutational multivariate analysis of variance (PERMANOVA) in the R package vegan^67,69,70^. Potentially confounding variables were listed first in the model, with the variable of interest listed last. The dispersion assumption for PERMANOVA, PERMDISP, was tested for significant values using the vegan package^69–71^. Welch’s t-tests were used to examine body condition variation (measured by weight:wing ratio) between groups with and without detected virulence factors. Kruskal Wallis tests were used to assess significant differences between relative abundances of metabolic pathways and groups from the WGS dataset, and false discovery correction was applied with the Benjamini–Hochberg method^72^.

### Ethical approval

Sampling procedures and sample export were approved by the University of St. Louis´s Institutional Animal Care and Use Committee (Protocol #1211796), USDA (Permit #47418), Galapagos National Park Directorate (#PC-05-18), the Agency for the Regulation and Control of Biosecurity and Quarantine for Galapagos (#ABG-CT-2019-0019-O), and the Ecuadorian Ministry of the Environment and Water (Contract #MAE-DNB-CM-2016-0043, Export Authorization #128-2019-EXP-CM-MBI-DNB/MA).

## Results

**Figure 1:**
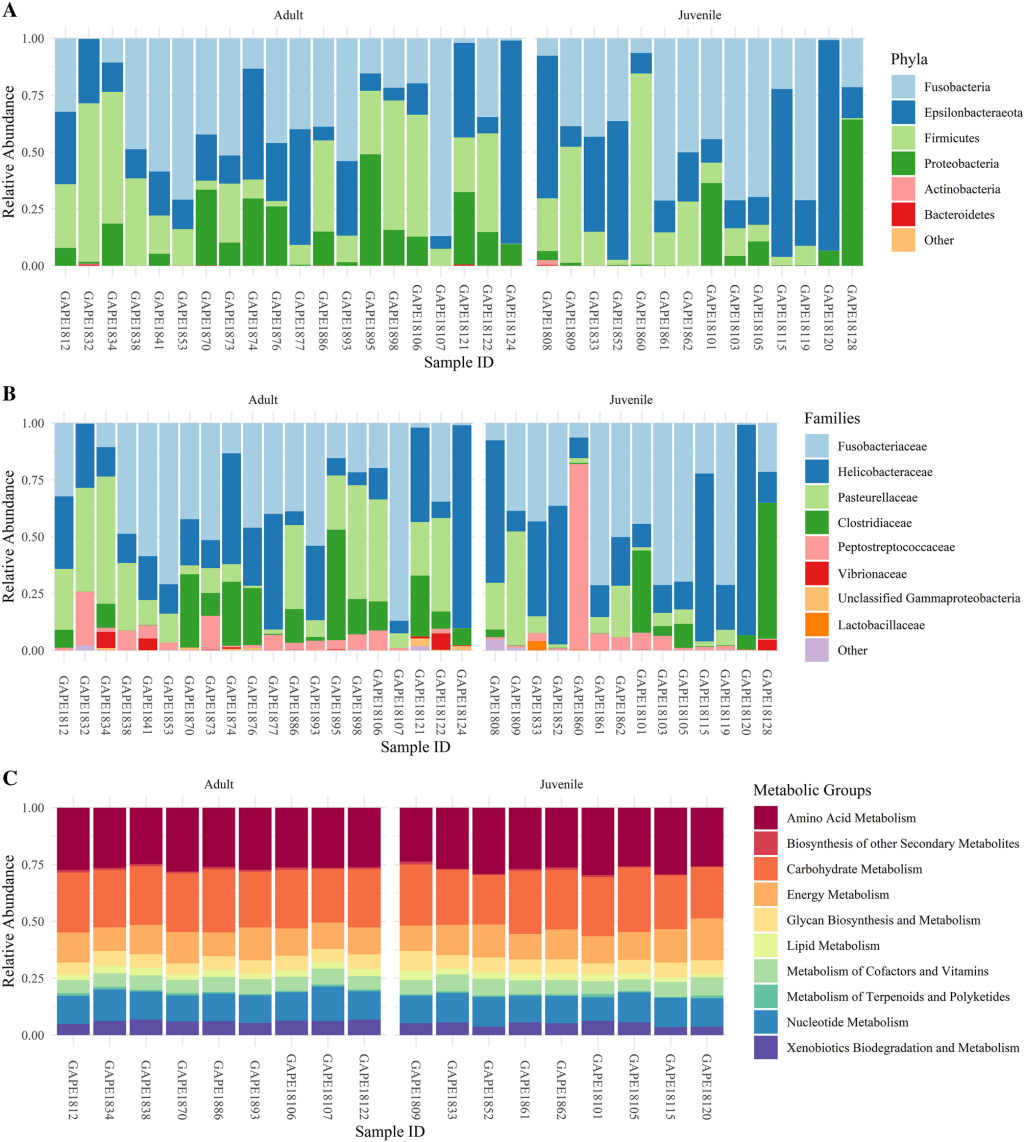
Bacterial phyla and families detected in 16S rRNA sequencing data (**A**, **B**), and metabolic KEGG pathway groups computed from shotgun sequencing data (**C**). Figure created in R using tidyverse (v. 1.3.1)^96^ and cowplot (v. 1.1.1)^97^.

The primary bacterial phyla found in the Galapagos penguin fecal samples were Fusobacteria, Epsilonbacteraeota, Firmicutes, and Proteobacteria, and the most common families were Fusobacteriaceae, Helicobacteraceae, Clostridiaceae, Pasteurellaceae, and Peptostreptococcaceae (Fig. 1). WGS samples were profiled functionally using level II of the KEGG pathway classification hierarchy. Most identified KEGG pathways were involved in metabolic activity, primarily amino acid metabolism, carbohydrate metabolism, energy metabolism, and nucleotide metabolism (Fig. 1).

**Figure 2:**
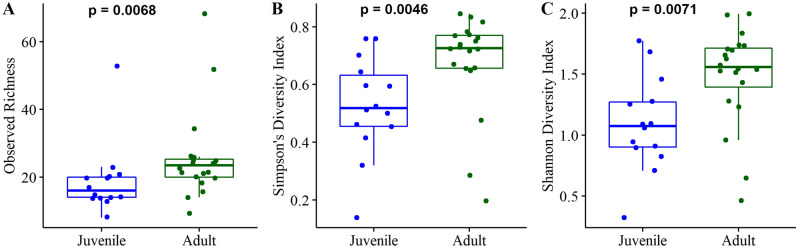
Alpha diversity was significantly lower in juveniles compared to adults when measured as observed richness (**A**), Simpson’s Diversity Index (**B**), and Shannon Diversity Index (**C**). Figure created in R using tidyverse (v. 1.3.1)^96^, cowplot (v. 1.1.1)^97^, and ggpubr (v. 0.4.0)^98^.

Alpha and beta diversity were calculated to assess diversity within and between communities, respectively. Overall, alpha diversity was low; no raw sample exceeded 100 identified OTUs and most contained fewer than 50 OTUs. Alpha diversity was significantly lower in juveniles compared to adults in each of the three diversity measures used (Fig. 2, observed richness *P* = 0.0068, Simpson’s *P* = 0.0046, Shannon *P* = 0.0071). Beta diversity significantly differed between age classes after controlling for location and sex in the model (PERMANOVA, R^2^ = 0.06427, *P* = 0.019, Supplementary Information; PERMDISP, *P* = 0.816). Adult penguins did not vary by sex when comparing either alpha or beta diversity. No functional differences based on age or sex were apparent in the WGS dataset with either metabolic pathways or protein families.

We also examined the importance of location for microbiome composition. Due to the uneven sample distribution across sites, single samples from El Muñeco (at the northern end of Isabela Island) and Playa Perros (near Puerto Pajas) were first excluded. The remaining three sites—Caleta Iguana, Puerto Pajas, and Marielas—represent the largest penguin colonies. Caleta Iguana is only represented by four samples following filtering steps, constraining our ability to compare between all three sites.

**Figure 3:**
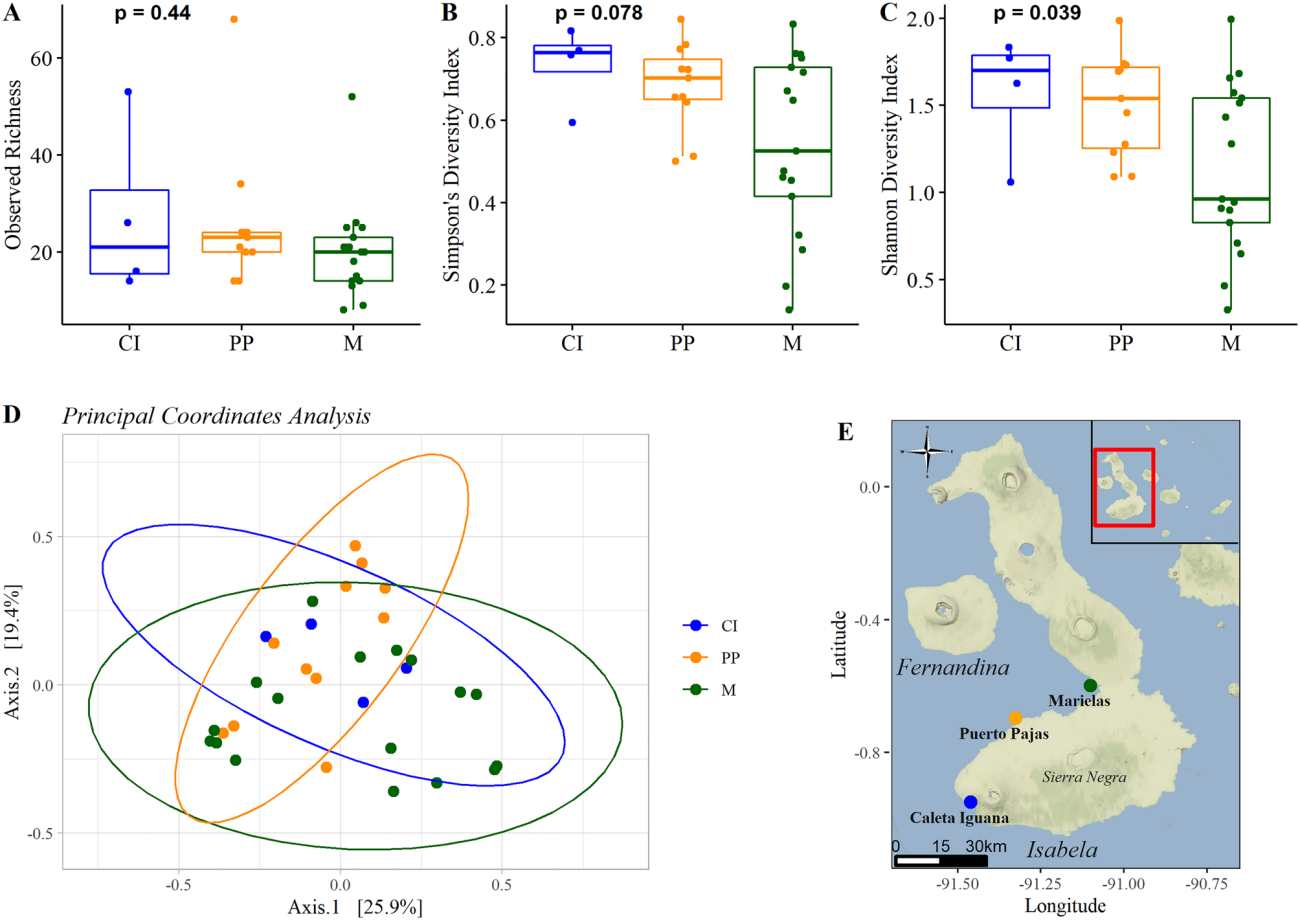
Alpha and beta diversity showed minor variation between samples from the three primary sampling sites on Isabela Island: Caleta Iguana (CI), Puerto Pajas (PP), and Marielas (M). Observed richness and Simpson’s Index were similar between sites (**A**, **B**), but Shannon Diversity Index significantly differed (**C**). Principal Coordinates Analysis illustrated slight clustering between locations (**D**). Sample sites are shown along the western coast of Isabela Island, with the Galapagos Archipelago in the map inset (**E**). Figure created in R using tidyverse (v. 1.3.1)^96^, phyloseq (v. 1.40.0)^67^, cowplot (v. 1.1.1)^97^, ggpubr (v. 0.4.0)^98^, ggmap (v. 3.0.0)^99^, ggsn (v. 0.5.0)^100^, and ggrepel^101^ (v. 0.9.1).

Slight clustering by location was apparent using Principal Coordinates Analysis (PCoA) (Fig. 3). Compared to the more exposed sample sites, Marielas was slightly different (PERMANOVA, 999 permutations, R^2^ = 0.05970, *P* = 0.038, Supplementary Information; PERMDISP, *P* = 0.293). However, when the sites were limited to Marielas and Puerto Pajas there was no significant difference (PERMANOVA, 999 permutations, R^2^ = 0.06588, *P* = 0.061, Supplementary Information). Shannon Diversity Index values significantly differed between the three sites (*P* = 0.03857), with the lowest Shannon Diversity seen at Marielas, but observed richness and Simpson’s Index did not significantly vary (Fig. 3).

### Antibiotic resistance screening

Screening shotgun sequencing reads from each sample with the CARD database revealed a total of nine putative antibiotic resistance genes (Table 1). Two resistance genes corresponding to *Helicobacter pylori* reference genomes were particularly widespread, occurring in almost all samples (19/20); these genes confer resistance to Tetracyclines and Macrolides. Genes resistant to Aminoglycosides and Lincosamides were also common in this small sample set, and one Peptide-resistant gene corresponding to *C. perfringens* SM101 was found in six samples.

**Table 1:** Antibiotic resistance genes detected in Galapagos penguin samples.

CARD Accession	Function	# Samples	Reference Genome	Antibiotic Class
ARO:3,003,510	*Helicobacter pylori* 16S rRNA mutation conferring resistance to tetracycline	19	*Helicobacter pylori* 26,695	Tetracycline
ARO:3,004,134	*Helicobacter pylori* 23S rRNA with mutation conferring resistance to clarithromycin	19	*Helicobacter pylori*	Macrolide
ARO:3,003,493	*Pasteurella multocida* 16S rRNA mutation conferring resistance to spectinomycin	9	*Pasteurella multocida* 36,950	Aminoglycoside
ARO:3,004,149	*Escherichia coli* 23S rRNA with mutation conferring resistance to clindamycin	9	*Escherichia coli* CFT073	Lincosamide
ARO:3,003,773	*Clostridium perfringens* mprF	6	*Clostridium perfringens* SM101	Peptide
ARO:3,001,219	*Clostridium difficile* EF-Tu mutants conferring resistance to elfamycin	6	*Clostridium difficile*	Elfamycin
ARO:3,003,512	*Salmonella enterica* serovar Typhimurium 16S rRNA mutation in the rrsD gene conferring resistance to spectinomycin	5	*Salmonella enterica* subsp. *salamae*	Aminoglycoside
ARO:3,004,058	*Staphylococcus aureus* 23S rRNA with mutation conferring resistance to linezolid	1	*Staphylococcus aureus*	Oxazolidinone
ARO:3,003,403	*Escherichia coli* 16S rRNA mutation in the rrsB gene conferring resistance to paromomycin	1	*Escherichia coli* K-12	Aminoglycoside

### Pathogen screening

WGS samples were screened for virulence factors using three tools with two databases: PATRIC (VFDB), HUMAnN 3.0 (Pfam), and MetaErg (Pfam). Virulence factors across all three tools were associated with a single bacterium, *Clostridium perfringens.* Most of the reference genome matches from the VFDB were from strain 13, classified as type A (Table 2). *C. perfringens* virulence factors from the VFDB were detected in 12/20 penguins, though the taxonomic search using Kraken 2 showed the presence of the bacterium in an additional seven penguins (19/20). The virulence-associated BrkB protein family was also classified with *C. perfringens* in both HUMAnN 3.0 and MetaErg (BrkB Pfam accession: PF0361), though to varying degrees—read-based HUMAnN 3.0 detected BrkB with *C. perfringens* in 8/20 samples, while contig-based MetaErg detected it in 10/20 samples. All eight of the samples with virulence factors detected by HUMAnN 3.0 were also highlighted by MetaErg, and all ten of the samples with virulence factors detected by MetaErg were also found by PATRIC.

**Table 2:** *Clostridium perfringens* virulence factors detected in PATRIC.

Template	Gene	Product	Virulence factor	# Samples	Reference Genome
VFDB|VFG002274	*plc*	Phospholipase C	alpha-toxin	9	*C. perfringens* str. 13
VFDB|VFG002277	*nagH*	Hyaluronidase	mu-toxin	8	*C. perfringens* str. 13
VFDB|VFG002276	*colA*	Collagenase	kappa-toxin	7	*C. perfringens* str. 13
VFDB|VFG002284	*nanJ*	Exo-alpha-sialidase	sialidase	7	*C. perfringens* str. 13
VFDB|VFG002285	*nanH*	Sialidase	sialidase	7	*C. perfringens* ATCC 13124
VFDB|VFG002275	*pfoA*	Perfringolysin O	theta-toxin	6	*C. perfringens* str. 13
VFDB|VFG002282	*cloSI*	Alpha-clostripain	alpha-clostripain	6	*C. perfringens* str. 13
VFDB|VFG002283	*nanI*	Exo-alpha-sialidase	sialidase	6	*C. perfringens* str. 13
VFDB|VFG002278	*nagI*	Hyaluronidase	mu-toxin	6	*C. perfringens* str. 13
VFDB|VFG002279	*nagJ*	Hyaluronidase	mu-toxin	6	*C. perfringens* str. 13
VFDB|VFG002280	*nagK*	Hyaluronidase	mu-toxin	5	*C. perfringens* str. 13
VFDB|VFG002281	*nagL*	Hyaluronidase	mu-toxin	5	*C. perfringens* str. 13
VFDB|VFG002286	*cpe*	Enterotoxin Cpe	CPE (*C. perfringens* enterotoxin)	2	*C. perfringens* SM101

Additional bacterial taxa were associated with virulence factors in Pfam, though none appeared in the VFDB search. *Cetobacterium ceti, Clostridium baratii, Clostridium thermobutyricum, Paeniclostridium sordellii,* and *Photobacterium damselae* were detected with virulence-associated protein families by both HUMAnN 3.0 and MetaErg. The contig-based approach, MetaErg, found an additional 28 virulence-associated species; the most common were *Helicobacter brantae*, *Gallibacterium anatis, Helicobacter* sp. 002,287,135*, Helicobacter* sp. 001,693,335, and *Fusobacterium* sp. 900,015,295. Most taxa were associated with the Pfam virulence factor BrkB, but others matched with a haemolysin (SMP_2), virulence-associated protein E, or virulence protein RhuM family, among others.

**Figure 4:**
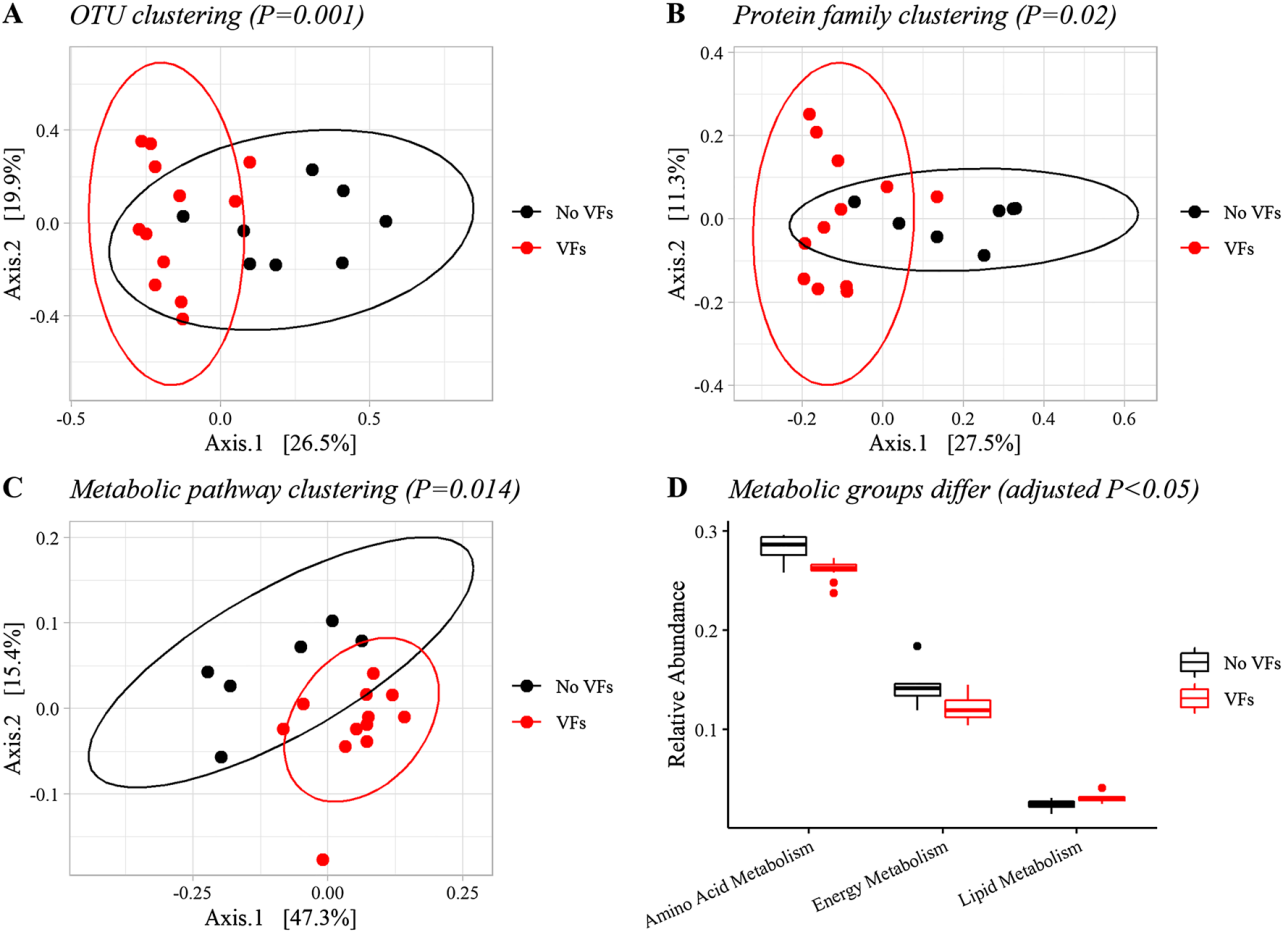
The penguin microbiome varied with *C. perfringens* virulence factors (VFs). Principal Coordinates Analysis showed distinct clustering based on OTUs from 16S rRNA gene sequencing data (**A**), protein families from shotgun sequencing data (**B**), and KEGG metabolic pathways from shotgun sequencing data (**C**) when separated by Jaccard distance and sorted by the presence of *C. perfringens* virulence factors from the VFDB. Relative abundance of three metabolic groups significantly varied in the presence of *C. perfringens* (**D**). Figure created in R using tidyverse (v. 1.3.1)^96^, phyloseq (v. 1.40.0)^67^, and cowplot (v. 1.1.1)^97^.

Since *C. perfringens* was the only bacterium consistently highlighted as a pathogen by all pipelines, the presence of *C. perfringens* virulence factors was examined as a potential structuring force for the microbial communities. Samples grouped by *C. perfringens* virulence factors from the VFDB revealed signals of dysbiosis (Fig. 4). Beta diversity was calculated from protein families and from metabolic pathway abundances, and in both cases samples with virulence factors clustered away from samples without virulence factors on a PCoA. After controlling for location and including age and sex in the models, both protein families (PERMANOVA, 999 permutations, R^2^ = 0.14156, *P* = 0.020, Supplementary Information; PERMDISP, *P* = 0.076) and metabolic pathways (PERMANOVA, 999 permutations, R^2^ = 0.22465, *P* = 0.014, Supplementary Information; PERMDISP, *P* = 0.081) were significantly different based on the presence of *C. perfringens* virulence factors. Three metabolic groups and 17 metabolic KEGG pathways significantly differed between samples divided by virulence factors (adjusted *P*-values < 0.05). This pattern held true in the 16S dataset, revealing taxonomic clustering based on the presence of *C. perfringens* virulence factors (PERMANOVA, 999 permutations, R^2^ = 0.17409, *P* = 0.001; PERMDISP, *P* = 0.27, Supplementary Information). However, alpha diversity did not significantly vary between groups. No significant relationship was found between *C. perfringens* status with age, sex, location, or body condition.

## Discussion

Overall, our study indicates that developmental stage, location, and pathogen presence are structuring the gut microbiome in the Galapagos penguin. The taxonomic profile of this community was similar to previously published penguin microbiomes, though the gut microbiome of the Galapagos penguin was notably dominated by Fusobacteria and lacking in Bacteroidetes^22,73,74^. We found adult Galapagos penguins had significantly higher alpha diversity (observed richness, Shannon Diversity, and Simpson’s Index) in their gut microbiomes compared to juvenile penguins. Differences in foraging behaviors and movement between adults and juveniles may be an explanatory factor^31,32^. Non-breeding adults and juveniles tend to travel longer distances as they forage for schooling fish and crustaceans in shallow water along the shore, while breeding adults travel shorter distances and stay near the nesting sites^31,32^. However, juveniles may return to the nest area as they gradually learn how to forage, and adults occasionally demonstrate extended parental care during this learning period by feeding fully-fledged juveniles, complicating movement-based interpretations of the reduced microbial diversity found in juveniles^31,75^. Hormonal differences could also play a role since some adults were in breeding condition—this is known to increase alpha diversity in males in other avian species—but surprisingly, no sex-based differences were found^19^. The lack of sex-based differences may again be related to foraging habits, as males and females exhibit similar foraging behaviors and are likely exposed to similar dietary and environmental microbes^32^. It is important to understand how factors such as developmental stage can influence the avian microbiome, as this could indicate a varied degree of vulnerability to disease depending on host age.

Though location did not appear to be a strong force structuring the microbiome, both alpha and beta diversity showed some differences at the Marielas Islets compared to the other primary sampling sites. The similar environment along the coast of Isabela and the movement of penguins between sites are likely factors behind the general homogeneity of microbiomes between locations^31,32^. The small differences observed may be explained by unique diets between sites or exposure to different environmental microbes. While sociality can also influence microbiomes, determining relatedness or pair bonds within the sample set was beyond the scope of this study^76^. Perhaps significantly, the Sierra Negra shield volcano on Isabela Island was erupting during the 2018 sampling trip. Lava flowed down the volcano’s northwestern flank and reached the sea near the Marielas sampling site^77^. This contributed to warmer water temperatures at that site and likely altered pH levels as well^78^. Changes in pH can influence aquatic microbial communities, and this proximity to volcanic activity may have led to the slight variation in microbial signatures found between sites^79^. Differing amounts of environmental heavy metal may also play a role, as this is known to alter microbiome compositions in some systems—a previous study found variation in heavy metal concentrations in Galapagos penguin feathers from different sites, with significantly higher levels of lead in feathers from Marielas^80,81^. An additional factor may be the relatively exposed position of the southern sites compared to the more sheltered location of the Marielas away from the primary current.

We determined that the putative resistome for this species contains at least nine resistance genes. Two resistance genes associated with *Helicobacter pylori* were almost ubiquitous, detected in all but one penguin. Antibiotic resistant genes have been previously found in the Galapagos Islands in marine water, tortoise feces, and both land iguana and marine iguana feces, but this is the first time to our knowledge that they have been detected in Galapagos penguins^9,42,43^. Some antibiotic resistance occurs naturally, in areas as remote as Antarctica, and resistance genes could potentially be found in Galapagos even in the absence of human activity^82^. However, the increasing amount of antibiotic resistance found in the wild is broadly attributed to selection from the heavy use of antibiotics in modern agricultural and clinical settings^83^. In one example of likely anthropogenic effects, a study in Galapagos found that proximity to humans (e.g. ports, towns) was generally associated with the antibiotic resistance found in seawater and reptile feces, with increased resistance detected in most populated areas and no resistance detected at certain isolated sites^9^.

The exchange of antibiotic resistance genes happens readily among bacteria through horizontal gene transfer, making it challenging to prevent resistance genes from spreading. Antibiotic resistance is found at high levels in bacteria from human waste, and even after waste treatment sewage remains a potent source of antibiotics or resistance genes^84^. Resistance genes can even be transferred by wildlife across large distances, such as through resident bacterial flora of migratory birds^82^. In Galapagos, sewage contamination from towns and boats is a likely way in which antibiotics and/or bacteria with resistance genes could be introduced into the environment (which can also be detrimental to human health), though resistance genes may also arrive from other sources^42^. Increased wastewater control is thus an essential factor to limit the spread of antibiotic resistance in wild communities. Finding antibiotic resistance consistently in the penguin samples indicates that it is present even in more isolated areas along Isabela Island, emphasizing a need for further investigation into the extent of resistance genes associated with anthropogenic activity (such as cruise ships) and whether wastewater management changes should be considered in the islands. Collecting water samples in parallel with penguin fecal samples in future studies would provide greater insight into where environmentally derived antibiotic resistance genes may have originated.

Finally, our pathogen screening highlighted a few potential enteric pathogens—*Clostridium perfringens, Paeniclostridium sordelli, Clostridium baratii, Gallibacterium anatis,* and *Photobacterium damselae* were among the most common bacteria linked with virulence-associated protein families in this microbiome*.* The class Clostridia contains some of the primary agents of enteric disease in birds, and *C. perfringens, P. sordellii* and *C. baratii* have all been associated with enteric disease^85,86^. *Gallibacterium anatis* (previously *Pasteurella anatis*) has also been implicated as a pathogen in several avian species and is considered an emerging poultry disease^87^. However, the detection of virulence-associated factors is by itself no guarantee that a microbe is actually pathogenic to the host, and microbes such as *Gallibacterium anatis* are also commonly found as members of the normal bacterial flora^88^. Further, several bacteria in the microbiome were associated with virulence proteins but are unlikely to be pathogenic to penguins—for example, *Photobacterium damselae* was linked to several different virulence-associated protein families in this microbiome, though this bacterium is recognized as a pathogen in taxa such as fish and marine mammals rather than birds^89^.

*Clostridium perfringens* was the only putative pathogen detected with the Virulence Factor Database in addition to the Protein Family Database (Pfam). *C. perfringens* is an extremely widespread bacterium and a normal member of many microbiomes, but it is also notorious for causing necrotic enteritis in poultry and other birds (as well as enteric diseases in humans, dogs, and a number of other taxa)^90,91^. In poultry, *C. perfringens* injects toxins into the intestines, resulting in intestinal lesions and a range of clinical signs including lethargy, loss of appetite, and mortality (though lethal cases may also occur without any observable symptoms)^86^. Outbreaks of *C. perfringens* leading to fatalities have also been documented in captive penguin populations^92^. The pathogenicity of the detected strain of *C. perfringens* in Galapagos penguins is unknown, and we notably did not detect the *netB* gene encoding a pore-forming toxin which is associated with most occurrences of necrotic enteritis in poultry^93^. However, the detected virulence factors correspond to type A and the genes *plc* and *cpe*, which are associated with toxin production and avian enteric disease^91^. Coupled with the apparent dominance of *C. perfringens* in the observed microbial communities and the strong structural changes observed in the presence of virulence factors, this suggests a level of pathogenicity at the time of sampling. While pathogen presence was not significantly associated with body condition in this study, a larger sample size would provide more conclusive results. Resampling the population is necessary to shed light on the role *C. perfringens* plays in this species’ microbiome.

The Galapagos penguin faces many threats, largely from anthropogenic sources. Climate change may lead to increased frequency of El Niño events, which would significantly increase the species’ odds of extinction^31,33,94^. Overfishing in the area surrounding the Galapagos National Park could influence food availability^31^. Introduced predators such as cats have been known to kill Galapagos penguins in the archipelago^31^. The low genetic variation found in these penguins may also increase their vulnerability to introduced diseases, and the small population size and limited range leave little room for species resilience in the event of an invading pathogen^30,31,35,38^. This study contributes to disease surveillance in this species and to understanding the degree of human influence reaching isolated penguin breeding sites. Future studies would benefit from parallel environmental microbiome samples, a larger sample size, and seasonal sampling to quantify temporal patterns of bacterial pathogens in this population.

## Conclusions

This work establishes a baseline microbiome for an endangered penguin, identifies two primary drivers of microbial community structure, and emphasizes the importance of minimizing interaction between wildlife and humans. Even in a site as remote and well-protected as the Galapagos Islands, human influence is still visible through factors such as antibiotic resistance genes. The human-inhabited islands also have some domesticated animals, which increases the possibility of disease spillover occurring between domestic and wild species—the apparent pathogenicity of *C. perfringens* found in Galapagos penguins is concerning when considering the proximity of the penguin population to domestic chickens^95^. Thus, monitoring and limiting anthropogenic effects on wildlife is critical to the continued long-term preservation of Galapagos endemic species.

## Supplementary Information

Supplementary Information.

## Data Availability

The raw sequencing files corresponding to this article are available in the NCBI Sequence Read Archive (SRA) repository under BioProject ID PRJNA794207 (https://www.ncbi.nlm.nih.gov/sra/PRJNA794207). R scripts and associated files are available at https://github.com/sd784/Rohrer_etal_GAPEmicrobiome.

## Abbreviations

PCR: Polymerase chain reaction
KMA: K-mer alignment
VF: Virulence factor
VFDB: Virulence factor database
CARD: Comprehensive antibiotic resistance database
PATRIC: Pathosystems resource integration center
Pfam: Protein family database
KEGG: Kyoto encyclopedia of genes and genomes
PCoA: Principal coordinates analysis
PERMANOVA: Permutational multivariate analysis of variance
