# Supplementary material for: Composition and function of the Galapagos penguin gut microbiome vary with age, location, and a putative bacterial pathogen
Source: Sci Rep. 2023 Apr 1;13:5358. doi: 10.1038/s41598-023-31826-y (PMC10067942; doi:10.1038/s41598-023-31826-y)
Supplement: Supplementary file 1 — Supplementary Information. [file 41598_2023_31826_MOESM1_ESM.docx]

**Supplementary Information**

Title: Composition and function of the Galapagos penguin gut microbiome vary with age, location, and a putative bacterial pathogen

Authors: Sage D. Rohrer, Gustavo Jiménez-Uzcátegui, Patricia G. Parker, & Lon M. Chubiz

**Table 1.** PERMANOVA results for age when controlling for location and sex (N = 34).

|  | **Df** | **SumOfSqs** | **R2** | **F** | **Pr(>F)** |
| --- | --- | --- | --- | --- | --- |
| **Location** | 4 | 1.3083 | 0.14933 | 1.3307 | 0.094 |
| **Sex** | 2 | 0.4995 | 0.05701 | 1.0161 | 0.413 |
| **Age** | 1 | 0.5631 | 0.06427 | 2.291 | 0.019* |
| **Residual** | 26 | 6.3903 | 0.7294 |  |  |
| **Total** | 33 | 8.7611 | 1 |  |  |

**Table 2.** PERMANOVA results for the primary sampling sites (Puerto Pajas vs. Marielas) when controlling for age and sex (N = 28). Caleta Iguana was excluded due to the low sample size.

|  | **Df** | **SumOfSqs** | **R2** | **F** | **Pr(>F)** |
| --- | --- | --- | --- | --- | --- |
| Age | 1 | 0.6376 | 0.08833 | 2.5839 | 0.013* |
| Sex | 2 | 0.4298 | 0.05954 | 0.8708 | 0.598 |
| Location | 1 | 0.4756 | 0.06588 | 1.9273 | 0.061 |
| Residual | 23 | 5.6754 | 0.78625 |  |  |
| Total | 27 | 7.2184 | 1 |  |  |

**Table 3.** PERMANOVA results for exposed sites, Puerto Pajas and Caleta Iguana, vs. the sheltered site, Marielas (N = 33). The single sample from El Muñeco was excluded.

|  | **Df** | **SumOfSqs** | **R2** | **F** | **Pr(>F)** |
| --- | --- | --- | --- | --- | --- |
| Age | 1 | 0.5714 | 0.06828 | 2.3489 | 0.023* |
| Site (Exposed vs. Unexposed) | 1 | 0.4996 | 0.0597 | 2.0538 | 0.038* |
| Residual | 30 | 7.2978 | 0.87202 |  |  |
| Total | 32 | 8.3689 | 1 |  |  |

**Table 4.** PERMANOVA results for the metabolic pathway dataset (whole genome sequencing data) between individuals with and without detected *C. perfringens* virulence factors (N = 18). Location was set as strata to control for potential confounding effects.

|  | **Df** | **SumOfSqs** | **R2** | **F** | **Pr(>F)** |
| --- | --- | --- | --- | --- | --- |
| Age | 1 | 0.03135 | 0.07268 | 1.4636 | 0.176 |
| Sex | 2 | 0.02465 | 0.05715 | 0.5755 | 0.88 |
| *C. perfringens* Virulence Factors | 1 | 0.0969 | 0.22465 | 4.5242 | 0.014 |
| Residual | 13 | 0.27843 | 0.64552 |  |  |
| Total | 17 | 0.43133 | 1 |  |  |

**Table 5.** PERMANOVA results for the protein family dataset (whole genome sequencing data) between individuals with and without detected *C. perfringens* virulence factors (N = 19). Location was set as strata to control for potential confounding effects.

|  | **Df** | **SumOfSqs** | **R2** | **F** | **Pr(>F)** |
| --- | --- | --- | --- | --- | --- |
| Age | 1 | 0.2518 | 0.05403 | 1.0779 | 0.342 |
| Sex | 2 | 0.2446 | 0.0525 | 0.5236 | 0.961 |
| *C. perfringens* Virulence Factors | 1 | 0.6597 | 0.14156 | 2.8241 | 0.02* |
| Residual | 15 | 3.5041 | 0.75191 |  |  |
| Total | 19 | 4.6603 | 1 |  |  |

**Table 6.** PERMANOVA results for 16S rRNA sequencing data divided between samples with and without detected *C. perfringens* virulence factors.

|  | **Df** | **SumOfSqs** | **R2** | **F** | **Pr(>F)** |
| --- | --- | --- | --- | --- | --- |
| Age | 1 | 0.4634 | 0.09733 | 2.271 | 0.017* |
| *C. perfringens* Virulence Factors | 1 | 0.8289 | 0.17409 | 4.0619 | 0.001*** |
| Residual | 17 | 3.4689 | 0.72858 |  |  |
| Total | 19 | 4.7612 | 1 |  |  |
